# Systematic narrative review of decision frameworks to select the appropriate modelling approaches for health economic evaluations

**DOI:** 10.1186/s13104-015-1202-0

**Published:** 2015-06-17

**Authors:** B Tsoi, D O’Reilly, J Jegathisawaran, J-E Tarride, G Blackhouse, R Goeree

**Affiliations:** Department of Clinical Epidemiology and Biostatistics, McMaster University, Hamilton, ON Canada; PATH Research Institute, St. Joseph’s Healthcare Hamilton, Hamilton, ON Canada; Centre for Evaluation of Medicines (CEM), St. Joseph’s Healthcare Hamilton, Hamilton, ON Canada

**Keywords:** Decision analysis, Health economic evaluation, Systematic review, Decision trees, State-transition models, Markov model, Microsimulation, Agent-based models, System dynamics, Compartmental models

## Abstract

**Background:**

In constructing or appraising a health economic model, an early consideration is whether the modelling approach selected is appropriate for the given decision problem. Frameworks and taxonomies that distinguish between modelling approaches can help make this decision more systematic and this study aims to identify and compare the decision frameworks proposed to date on this topic area.

**Methods:**

A systematic review was conducted to identify frameworks from peer-reviewed and grey literature sources. The following databases were searched: OVID Medline and EMBASE; Wiley’s Cochrane Library and Health Economic Evaluation Database; PubMed; and ProQuest.

**Results:**

Eight decision frameworks were identified, each focused on a different set of modelling approaches and employing a different collection of selection criterion. The selection criteria can be categorized as either: (i) structural features (i.e. technical elements that are factual in nature) or (ii) practical considerations (i.e. context-dependent attributes). The most commonly mentioned structural features were population resolution (i.e. aggregate vs. individual) and interactivity (i.e. static vs. dynamic). Furthermore, understanding the needs of the end-users and stakeholders was frequently incorporated as a criterion within these frameworks.

**Conclusions:**

There is presently no universally-accepted framework for selecting an economic modelling approach. Rather, each highlights different criteria that may be of importance when determining whether a modelling approach is appropriate. Further discussion is thus necessary as the modelling approach selected will impact the validity of the underlying economic model and have downstream implications on its efficiency, transparency and relevance to decision-makers.

**Electronic supplementary material:**

The online version of this article (doi:10.1186/s13104-015-1202-0) contains supplementary material, which is available to authorized users.

## Background

The use of decision-analytic modelling to estimate the cost-effectiveness of health care interventions is becoming widespread to inform health policy decision-making. A model, referred to in this article, is defined as the use of analytical methodology to quantitatively compare health technologies. Models may have a range of uses including extrapolating from primary data sources and transferring results from one jurisdiction to another [1]. By incorporating event probabilities, resource utilization, costs and patient outcomes, a model synthesizes the data to identify the best option for decision-makers.

However, with the growing reliance on economic evaluations to support decision-making, concerns have risen on the validity, reliability and comparability of the results generated from such models [1]. To respond to these criticisms, the research community has focused considerable efforts in setting best practice guidelines for the development and conduct of health economic models. This is evident from the guidelines published by respective health technology assessment (HTA) agencies (e.g. Canadian Agency for Drugs and Technologies in Health (CADTH) [2]) and from non-profit research organizations (e.g. International Society For Pharmacoeconomics and Outcomes Research (ISPOR) [3–5]).

With the growing diversity of modelling approaches available (Table 1), a particular question is the relative merits of each approach in health economic modelling. Historically and still to date, decision trees and Markov cohort models are the most commonly used approaches in economic evaluation. However, due to their limitations, awareness has grown on alternative modelling approaches. Guidance documents recommend transparent reporting of a modeller’s rationale for selecting a model type, although it may not always be clear which approach would be most suitable for a given decision problem. This is an important issue since each approach can introduce constraints to a model’s development and its conceptualization in terms of what elements can be captured and the ease to which they can be incorporated into the model [6]. This may lead to a different focus on the decision problem and, thereby, generate conflicting results and diverging policy recommendations [7].

**Table 1:** Description of modelling approaches employed in health economic evaluation

Model approach	Description (key terminology italicized)
Decision tree	Decision trees embody the central paradigm of decision analysis. Events in the tree are typically arranged in temporal order from left to right. Decisions are broken down into three components: (i) *Decision node* decision point between competing strategies (ii) *Chance node* consequence to a given decision. Typically indicates point where two or more alternative chance events for a patient are possible. May contain sequential chance events (iii) *Terminal node* Terminal branch, representing the value of a particular strategy *Branches* connect the nodes and represent the pathways through the tree. At each chance node, the probabilities of each consequence will determine the proportion of patients progressing down each unique path Consequences such as costs and effects of events and decisions may be attributed at each chance node of the tree or accumulated at the terminal nodes. The expected effect and/or costs associated with each treatment option or branch is estimated by ‘*rolling*’ *back* the tree whereby a weighted average of the value of all branches emanating from a decision node is calculated
Markov cohort model	Markov cohort models describe the transition of patients as they move through health states over time. *Health states* are mutually exclusive events, representing the entirety of the disease process and patients are assumed to be in one of a finite number of health states (known as the *unitary state requirement*). Patients within the same health state are assumed homogeneous Movement between health states are governed by *transition probabilities* that occur only once per *Markov cycle* (i.e. a defined time period). The transition probabilities depend only on the starting state and not on any of the previous health states (i.e. memoryless assumption) The model is run over many cycles to build a profile of how many patients are in each state of the model over time Estimates of costs and health outcomes are attached to the states within the model. *Cycle sum* are calculated as the weighted average of the proportion of a cohort in a health state multiplied by the value for that particular health state, summing across all health states. Expected costs and QALYs are then calculated by summing all cycle sums over the model’s time horizon
Markov microsimulation	Markov microsimulation simulates individual patients over time. As individuals are modelled separately, microsimulation can store information as to what has happened to the individual (i.e. memory). Similarly, as individuals are modelled, there is no need to assume homogeneity between patients. The unitary state requirement remains as patients can only be in one of a finite number of health states during each cycle. Transitions govern patient prognosis and are calculated by model parameters that reflect actual event/transition rates and may be conditional on previous and current risk factors and historical outcomes. Transitions occur only once per cycle Consequences such as costs and effects of events are attributed to health states and are summed over each cycle. Each patient has their own respective costs and outcome following a run through the model and the expected costs and QALYs can be calculated as the average from a large number of patients that have gone through the model
Discrete event simulation	Discrete event simulation describes the flow of *entities* through the treatment system. Entities are objects, such as individuals, that may interact indirectly with other entities within the system when waiting for resources to become available. Entities may be given *attributes*, such as characteristics or memory, which may influence their route through the simulation and/or the length of time between events. Another important concept is *resources*, representing an object that provides service to a dynamic entity Life (and disease) histories of individuals are simulated one-by-one or simultaneously. If simulated simultaneously, one can model entity interactions or resource competition, thereby, explicitly embedding the effects of *queues* Consequences such as costs and effects can be attached to events, resource use or time with a particular condition
Agent-based model	This approach focuses on the *agent*. Agents are aware of their state and follow decision rules on how to communicate and interact with other agents or their environment. Agents are flexible as they may adapt over time, learn from experience and/or exist within a hierarchical structure. From simple rules governing individual actions and communication, complex behaviour may emerge As agents exist within a *network*, social network analysis may be used to examine interventions that impact inter-agent relationships and communication. It further provides a means for spatial considerations and can examine interventions that have a geographic impact Consequences such as costs and effects can be attributed to the events or patient attributes
System dynamics model	The *causal loop diagram* provides a qualitative visualization of a system’s structure. Its basic building block is the *feedback loop*, describing change at one point within a system that triggers a cascading series of changes that ripple through and eventually returns in some form to either reinforce or push back against that original change. Complex behaviour may emerge from the interaction of multiple feedback loops The system dynamics model is quantified by stock and flow diagrams. As per its name, these diagrams consist of two main variable types: *stocks* (also referred to as levels or state or accumulations) and *flows* (i.e. rates at which stocks are either drained or replenished). Movement between stocks is defined by the rate of flow and, together, a system’s behaviour may be described through a set of differential equations Costs and outcomes may be attributed to the time-in-stocks or movements between stocks that are continuously updated
Compartmental model	Compartmental models are historically used to model the epidemiology of infectious disease. The population is divided into various *compartments*, representing their average state. Individuals within a single compartment are considered homogeneous. Most commonly, it contains compartments of the population whom are at different stages of the illness (e.g. susceptible, exposed, infectious, recovered).

To provide guidance on how to select a particular modelling approach, frameworks have emerged that categorize and distinguish between them. However, no attempt has been made to compare and contrast these frameworks. The purpose of this paper is therefore to conduct a systematic literature review to identify and critically appraise these published frameworks.

## Methods

### Search methods

A literature search was performed for articles published up to January 21, 2014 with the following bibliographic databases searched: OVID Medline (1946-present; In-Process & Other Non-Indexed Citations) and EMBASE (1996-present); Wiley’s Cochrane Library (Issue 1 of 12, Apr 2014) and Health Economic Evaluation Database; PubMed (for non-Medline records); and ProQuest Dissertations. Controlled vocabulary terms, such as the National Library of Medicine’s Medical Subject Headings (MeSH), and keywords were used to construct the search strategy (Additional file 1, Additional file 2). The electronic search was supplemented by cross-checking the bibliographies of relevant publications and grey literature searches (e.g. working papers, commissioned reports, policy documents, websites).

### Selection of relevant articles

Records were screened for inclusion based on the pre-defined criteria presented in Additional file 3. To be included, a paper had to describe, in whole or in part, a decision framework (e.g. algorithm, taxonomy) on how to select between economic modelling approaches in the context of health care policy decision-making. Studies were limited to those published in English.

The titles and abstracts of the records identified from the bibliographic search were initially screened for relevance by one reviewer (B.T.) with a 50% random check conducted by a second independent reviewer (J.J.). If either reviewers identified a citation as being potentially relevant, its full-text was obtained. In the second phase of screening, one reviewer (B.T.) assessed the full-text version of all included articles, with a second independent reviewer (J.J.) completing a 50% random sample. Any discrepancies at this stage were resolved through discussion and consensus.

### Data extraction

A standardized data abstraction form was developed to extract data from the relevant studies. The form captured: bibliographic information (e.g. author, year); framework type (e.g. flow-chart, table); framework description, including its selection criteria; and the main conclusions. Their evolution and history, if discussed, was further noted.

The selection criteria specific to each decision framework were identified. These had to be present within the framework; criteria that were simply mentioned in the paper but not explicitly incorporated into the framework were excluded. These criteria were separated into either structural features or practical considerations. Structural features were defined as those relating to principles or theories behind a model. These are the technical elements that lay bare the intricacies of modelling concepts and the nature of the decision problem will dictate the structural features desired within a model. Practical considerations are defined as elements that impact the effectiveness or feasibility of developing and constructing a model and are, to a degree, context-dependent.

### Data analysis

Data were analyzed and synthesized with the intent to:

i. Understand the evolution of the frameworks;
ii. Tabulate and identify the frequency to which selection criteria were discussed across these frameworks;
iii. Evaluate the extent to which the frameworks agree or disagree on the structural features specific to each modelling approach.

## Results

**Figure 1:**
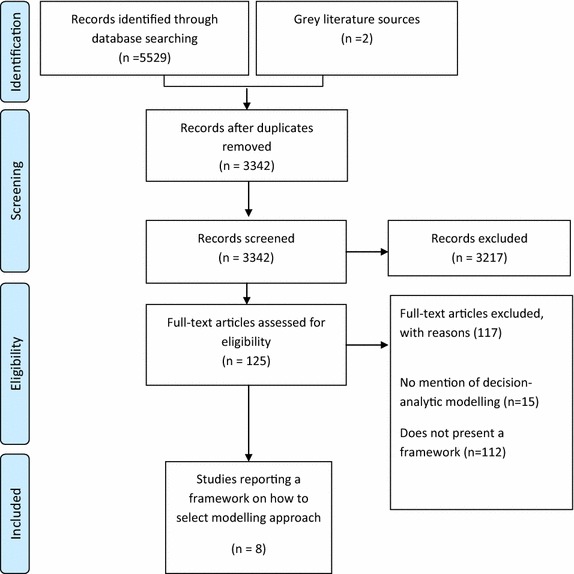
PRISMA diagram of literature search for articles on decision frameworks to select the appropriate modelling approach.

Of the 3,342 unique publications identified from the literature search, eight met the full inclusion criteria (Figure 1). Most studies were excluded either because it made no mention to decision-analytic modelling or it did not present a selection framework to guide the choice between modelling approaches. Overall, the agreement between independent reviewer for study inclusion was considered moderate (Cohen’s kappa 0.60).

Table 2 provides an overview of the decision frameworks in terms of the country of publication, the framework’s focus and the modelling approaches that were covered. All decision frameworks were published in the past 10 years with two specific to infectious diseases [6, 8] and the remainder being generic/non-disease specific [7, 9–13]. Each framework covered different model types, although all of them involved a decision between a traditional modelling approach (i.e. decision tree and Markov cohort model) and one or more alternative approaches (e.g. discrete event simulation; agent-based model; system dynamics).

**Table 2:** Overview of the decision framework and the modelling approaches covered within the respective frameworks

References	Country	Generic or disease-specific framework	Modelling approaches mentioned within the frameworks							Notes (i.e. relating to semantics and terminology)
			Decision tree	Markov cohort model	Markov microsimulation	Discrete event simulation	Agent-based models	System dynamics	Compartmental models	
Jit and Brisson [6]	United Kingdom; Canada	Infectious-disease	X	X					X	Static model aggregates decision tree and Markov cohort models; dynamic model refers specifically to compartmental models
Kim [8]	USA	Infectious disease	X	X	X	X	X	X	X	System dynamics models further separated into *discrete difference equation, ordinary differential equations* and *partial differential equations models*
Heeg [7]	The Netherlands	Generic	X	X	X	X				Markov microsimulation referred to as *first*-*order Markov model*
Stahl [13]	USA	Generic	X	X		X	X	X		Markov model, in this framework, does not distinguish between Markov cohort models and Markov microsimulations
Chick [11]	France	Generic	X	X	X	X		X		System dynamics model separated into *finite difference model* and *ordinary differential equation* (ODE). Markov microsimulation referred to as *patient*-*level simulation* DES includes both discrete event simulation and patients evolve on discrete-time grid. Decision tree described in many ways including: decision tree, stochastic decision tree, and stochastic decision tree with covariates
Cooper [12]	United Kingdom	Generic	X	X		X				
Brennan [10]	United Kingdom	Generic	X	X	X	X		X		Markov model also referred to as simulated Markov model. Markov microsimulation separated into *simulated patient*-*level Markov model* and *individual event history model*. System dynamics model also referred to as *Markov chain models*
Barton [9]	United Kingdom	Generic	X	X	X	X		X		Markov microsimulation referred to as *individual sampling model*

Decision frameworks were visually represented by flow charts [6, 9, 12, 13], radar graphs [7], or tables [8, 10, 11] and Table 3 further details the selection criteria that were considered within each framework. The definition of common structural features and practical consideration that were identified from this review of frameworks are presented in Table 4. The structural elements include: the resolution of the population; the capture of first-order uncertainty; the nature of the interactions; the handling of resource constraint; and the dimension of time. From Table 3, it was found that the most common structural features considered amongst these frameworks were interactivity (i.e. static vs. dynamic) and population resolution (i.e. aggregate or individual) (n = 6/8; 75%), followed by how time is handled (n = 4/8; 50%) (Table 3). Practical considerations (Table 4b) were explicitly included within most flowcharts and the most common practical consideration were the end-user requirements and simplicity (n = 3/8; 37.5%) (Table 3).

**Table 3:** Summary of the decision criteria (i.e. structural features and practical considerations) considered within each decision framework

References	Type of framework	Framework elements										
		Structural features						Practical considerations				
		Population resolution	First order uncertainty	Interactivity	Resource constraints	Dimension of Time	Other	Time	End-user requirement	Simplicity	Validity	Other
(a) Generic decision frameworks												
Heeg [7]	Radar graph	X	X		X	X	Memory	X			X	Experience
Stahl [13]	Flow diagram	X		X	X	X	Agent autonomy Spatial consideration		X			
Chick [11]	Table	X	X			X	Expected value					
Cooper [12]	Flow diagram			X^a^	X^a^		Modelled duration Recurrence Aggregation of cohort^b^	X	X	X	X	Model error
Brennan [10]	Table	X	X	X^a^	X^a^	X	Expected value					
Barton [9]	Flow diagram	X		X^a^	X^a^					X	X	
(b) Decision frameworks specific to infectious disease modelling												
Jit and Brisson [6]	Flow diagram			X								
Kim [8]	Table	X	X	X								

^a^Interaction, as defined in this framework, includes both interaction between individuals or constraints in resources that affect individuals.

^b^Aggregation of cohort refers to whether a single or multiple cohort of patients are modelled. It is commonly referred to whether the population is open (i.e. new individuals can enter model) or closed (i.e. no new additions are made in the model).

**Table 4:** Nomenclature and definition of commonly-mentioned decision criterion for selecting a modelling approach

(a) Structural features	Question to differentiate between attributes:	Typical classification	Definition
Population resolution	What level is the model arising?	Aggregate (may also be referred to as cohort)	The model is at a macro-level with a population aggregated and run through the model together. Variables represent population averages [8]. Relies on a homogeneity assumption that individuals within a particular health state are homogeneous [10, 12]. To incorporate individual factors or memories into the model, separate health states are required [10, 12]. Interactions are also modelled at an aggregate level
		Individual	The model is at a micro-level with individuals going through the model separately [8, 10, 13]. This easily incorporates individual factors and memory. Patient characteristics may be retained as continuous variables [4] Permits exploration of first-order uncertainty
First order uncertainty [14]	To what extent is the model capable of incorporating and analysing patient-level variability within its structure?	Deterministic	No variability in the outcomes between identical patients. Within a given sample of patients, individuals facing the same probabilities and outcomes will experience the effects of a disease or intervention identically
		Stochastic	Permits random variability in outcomes between identical patients as there exists uncertainty in patient-level outcomes that is entirely due to chance. Within a given sample of patients, individuals facing the same probabilities and outcomes will experience the effects of a disease or intervention differently. This can be perceived as a form of random error and, with increased sample size, the extent of this uncertainty can be reduced
Interactivity	Are actors in a model or the overall system independent?	Static/independent	No interaction present between or within actors as each actor is independent and no interactions at the system level [9]
		Dynamic/dependent	Interaction exists between or within actors or at the level of the system. Feedback and interdependencies may exist within the modelled system [9]
Resource constraint	Are constrained resources or queuing important to the decision problem?	Unlimited	There exist no constraints in the system
		Constrained	Resource constraints has impacts on features within the model [13]
Dimension of time	How is time handled by the model?	Untimed	Time is not explicitly modelled. Another term used to describe this concept of time is “aggregate” as changes in time are not considered important to the model [13]
		Discrete	Time separated into discrete units with an event occurring during one of the discrete time steps [8, 13]. To handle simultaneous events, requires smaller fixed time intervals [10]
		Continuous	Time is continuous with an event occurring at any point in the continuum of time; thereby, permits modelling of multiple simultaneous events [8]

(b) Practical consideration	Definition
Data availability	The availability of the necessary data to populate the economic model [7]
End-user Requirement	This considers whether the model meets the need of its end-users and decision-makers. It is dependent on how well the model structure reflects and is able to capture all relevant aspects of the underlying reality and the corresponding uncertainties that exist [6, 10]. End user requirement may capture whether the modelling approach is considered acceptable and whether funding is present to support a particular project
Experience	The extent to which the modeller has accumulated knowledge and implementation skills to construct the model [7]
Model error	The degree of imprecision in the model that is deemed acceptable by either the modeller and/or its end-users [12]. Model error can either be systematic or unsystematic. Unsystematic error, synonymous to uncertainty, can be explored through the application of sensitivity analysis. The feasibility of conducting sensitivity analysis is dependent on the model structure and its underlying parameters
Modelling software availability	The accessibility of the necessary software(s) to construct and evaluate the model. Different software may support different modelling approaches and are associated with licensing fees. Softwares for health economic modelling include Microsoft Excel (for decision trees and Markov cohort models); Treeage (for decision tree, Markov cohort model and Markov microsimulation); Arena (for discrete-event simulation); Any Logic (for discrete-event simulation, agent-based model, system-dynamics and compartmental models); and Berkeley Madonna (for system-dynamics and compartmental models)
Simplicity	The degree of complexity in a model. This is essentially dependent on the size of the model (e.g. the number of states/transitions in state-transition models) and the number of parameters present [9]. Simpler models are more likely to be understood and accepted by stakeholders [12]
Time	This considers the speed of model development and captures several aspects including the time required to programme the model (building time), the time required to collect the necessary data to fill the model (data collection) and the time required to generate simulation results (simulation time) [7]
Transparency	The degree to which the end-user of the model can review the model structure, equations, parameter values and the underlying assumptions. This is considered important by modellers for two reasons: (i) to provide non-quantitative description of the model to those interested in understanding how a model works; and (ii) to provide technical information to those interested in evaluating a model at the higher level mathematical and programming detail, possibly with the interest to replicate the results. Transparency promotes an understanding on the model’s accuracy, limitation and potential application. This is deemed important to build trust and confidence in a model to the appropriate decision-makers [15]
Validity	The clinical representativeness of a model to the actual decision problem [7, 12]. This addresses how adequately a chosen modelling approach reflects and captures all relevant aspects of the underlying reality and the corresponding uncertainties that exist

Below, a narrative summary of each framework is presented. A copy of each decision framework can be further found in Additional file 4.

### Generic frameworks

The first paper within the health care field on this topic was by Barton et al. [9]. Based on the following four criteria, their flowchart assists in the selection between decision tree, Markov cohort model, Markov microsimulation, discrete event simulation and system dynamics: (i) interactivity—importance of capturing interaction between patients; (ii) population resolution—the necessity of individual-level modelling; (iii) validity—the adequacy of pathways represented by a decision tree; and (iv) simplicity—the number of states required in a Markov cohort model. The authors highlight the trade-off between simplicity and clinical validity. They recommend a more complex and computational-demanding model only if it provides a more accurate representation of the decision problem and leads to more valid results [9]. Simplification, according to the authors, may involve fixing one or more parameters in the model and two conditions may justify such a practice: when the results are robust to variation with that particular set of parameters or if the parameter is derived from good and accurate data.

Brennan et al. [10] have proposed a taxonomy table describing the relationships between modelling approaches according to their structural features. The columns in their taxonomy highlight the assumptions of population resolution; expected value/memory and first-level uncertainty while the rows describes the interaction between individuals and the handling of time. Each cell in the table lists the modelling approach with those corresponding structural features. Some of the model structures described in this taxonomy can be considered subclassification of specific modelling approaches. For instance, depending on the dimension of time and first-order uncertainty, system dynamics was separated into finite difference equation system dynamics, ordinary differential equation system dynamics, discrete time Markov chain model and continuous time Markov chain model. The authors state that the identification of health states and risk factors, and their underlying relationships should precede the selection of a modelling approach. If multiple approaches are suitable, the simplest model that accurately addresses the decision problem should be chosen with further consideration on practical factors such as software availability, implementation skills, time constraints and end-user requirements [10].

Chick [11] simplifies Brennan’s proposed taxonomy by removing the rows specific to the dimension of interactivity; thereby, reducing the subclassification of certain modelling approaches seen in Brennan et al’s original taxonomy (i.e. microsimulation, system dynamics). However, it remains unclear why, for one set of features (i.e. stochastic Markovian individual discrete time), the cell is empty in Chick’s framework and is not associated with any particular modelling approach [11]. For this particular set of features, Brennan et al’s described the model structure as: ‘discrete-time individual event history model’.

Similarly, Heeg et al. [7] adapts Brennan’s [10] framework. However, rather than using a taxonomy table, they displayed their framework as a radar diagram that ranks the relative ability of decision tree, Markov cohort model, discrete event simulation and Markov microsimulation in addressing a collection of selection criterion—including practical considerations. Each spoke on the radar diagram represents a particular selection criterion and modelling approaches that are better at addressing that criterion appear further away from the origin of the radar diagram. Their framework incorporates all of the technical features proposed by Brennan although different terminologies are employed: ‘randomness’ is now referred to as ‘variability’ (i.e. first order uncertainty) while ‘expected value’ is referred to as ‘memory’ [7]. An additional technical feature included is the interaction due to covariates and nonlinear associations between individual risk factors and outcomes. The following practical considerations were also included in their framework: time (i.e. to collect data, build and simulate the model); experience and validity (i.e. clinical representativeness) (Table 3) [7].

An independent framework developed by Cooper et al. [12] similarly intertwined practical and structural considerations (Table 3) to help guide the decision between Markov cohort model, decision tree and discrete event simulation. The authors state that the nature and the complexity of the disease, and the health care intervention, may influence which structural features to consider (e.g. interaction between individuals; queuing and resource constraints) [12]. Rather than considering population resolution explicitly as a structural feature, this framework mentioned the impact of dimensionality in terms of the differences in time required to build and conduct simulations between aggregate-level and individual-level models. Outside of their framework, the modeller’s experience and data availability were additional factors that, together, may impact the speed and the ease of model development. The authors recommend that the analysis should be built based on the simplest model that can adequately address the research question [12]. A unique trait in Cooper’s framework is that it recognizes that modelling may not always be possible, and further incorporates an ‘abandon’ scenario when it is futile to pursue modelling given the disconnect between practical constraints and the desired technical attributes (e.g. significant heterogeneity and/or when queuing or interaction between individuals is important). In such cases, when the practical elements and the structural features conflict, construction of a model should be stopped until such issues are resolved [12].

By moving through a series of decisions pertaining mainly to the desired structural features, Stahl’s [13] hierarchical flowchart filters the choice of modelling approaches down to one to two suitable ones. Similar to Cooper [12], Stahl also advocates that simplicity should be a guiding principle—referring to it as, ‘keep it simple stupid (KISS)’ with a model only as complex as necessary for the question(s) of interest [13].

### Infectious disease specific frameworks

Brennan et al’s framework [10] was modified by another group of researchers for the evaluation of vaccines. Models were categorized according to three structural features: population resolution; first-order uncertainty; and interactivity [8]. As the selection criteria are dichotomous, eight possible categories exist (n = 2^3^) although only six categories were linked to modelling approach(es) as some combinations were deemed unrealistic. Kim et al. further recommend that model choice should be based on not only the nature of the decision problem (e.g. research question, natural history and features of the disease) but on practical concerns such as data availability, an analyst’s experience and time [8].

The last framework, by Jit and Brisson [6], utilized a series of questions organized into a flowchart to highlight the key distinctions between static (referred to as cohort models) and dynamic models in the context of infectious disease modelling. According to the authors, infectious diseases have several complexities that make it unique compared to other illnesses: transmissibility (i.e. interaction between infected and susceptible individuals); natural immunity; and the epidemiology of the illness (i.e. an infection proceeds through several stages, such as: susceptibility, latency/incubation, infectious/symptomatic and recovery) [6]. These distinctions result in the need for dynamic modelling when the force of infection is not constant over time. Instances include if an intervention changes the profile of the infected individuals (e.g. increase pathogenicity or transmissibility by shifting the age profile of the disease) or induces selective evolution on a subset of the organisms (e.g. antibiotic resistance) [6].

### Consistency between decision frameworks

Given that several decision frameworks were identified, it was of interest to assess the concordance in the frameworks’ recommendations. To conduct this, the structural features were evaluated across frameworks to assess their consistency in how they categorize each modelling approach in terms of their structural traits. As previously mentioned, structural features are expected to remain the same across decision frameworks for each modelling approach since they are based on theories and facts.

Table 5 presents the degree to which the decision frameworks are consistent in how they classify the structural assumptions specific to each modelling approach. As only two frameworks included agent-based models, both agreed that it is an individual-level approach that can incorporate interactions. System dynamics was seen as an aggregate-level approach that could handle interactions. Amongst the frameworks that do discuss the mechanism of time, system dynamic was considered able to model at a discrete unit or continuously although their capacity for handling resource constraints has yet to be addressed. Markov microsimulations have been characterized by the majority of the frameworks as an individual-level approach with time handled discretely or continuously. Few frameworks have addressed first-order uncertainty and the capability of Markov microsimulations in handling resource constraints except for one that suggested that microsimulations can assume unlimited resources [7]. Disagreement between frameworks remained on whether it is capable of handling interaction. For discrete event simulation, of those that addressed resource constraints and first order uncertainty, they all agreed on its capacity to incorporate resource constraints and that it is stochastic. The majority considered discrete event simulation as being capable of handling interactions between patients. However, discrepancies lay on how to classify the resolution of such models. For compartmental models, only the features of population resolution, first order uncertainty and interactivity have been discussed so far with the sole agreement being that this approach can incorporate interactions (Table 5).

**Table 5:** Classification of structural elements, specific to each modelling approach, according to the decision frameworks

Modelling approach	Number of frameworks that include this modelling approach n (%^a^)	Structural assumptions n (%^b^) [reference]															
		Population resolution			First-order uncertainty			Interactivity			Resource constraints			Dimension of time			
		Not specified within framework	Classification		Not specified within framework	Classification		Not specified within framework	Classification		Not specified within framework	Classification		Not specified within framework	Classification		
			Cohort	Individual		Deterministic	Stochastic		Static/independent	Dynamic/dependent		Unlimited resources	Constrained resources		Untimed	Discrete	Continuous
Decision tree	8 (100)	3 (37.5) [6, 9, 12]	5 (62.5) [7, 8, 10, 11, 13]	3 (37.5) [10, 11, 13]	4 (50) [6, 9, 12, 13]	4 (50) [7, 8, 10, 11]	2 (25) [10, 11]	2 (25) [7, 11]	6 (75) [6, 8–10, 12, 13]	0	6 (75) [6, 8–11, 13]	2 (25) [7, 12]	0	4 (50) [6, 8, 9, 12]	4 (50) [7, 10, 11, 13]	0	0
Markov cohort model	8 (100)	3 (37.5) [6, 9, 12]	5 (62.5) [7, 8, 10, 11, 13]	0	4 (50) [6, 9, 12, 13]	3 (37.5) [7, 8, 10]	2 (25) [10, 11]	2 (25) [7, 11]	6 (75) [6, 8–10, 12, 13]	0	6 (75) [6, 8–11, 13]	2 (25) [7, 12]	0	4 (50) [6, 8, 9, 12]	0	4 (50) [7, 10, 11, 13]	1 (12.5) [13]
Microsimulation	6 (75)	1 (16.6) [9]	0	5 (83.3) [7, 8, 10, 11, 13]	3 (50) [9, 10, 13]	0	3 (50) [7, 8, 11]	2 (33.3) [7, 11]	4 (66.7) [8–10, 13]	2 (33.3) [8, 10]	5 (83.3) [8–11, 13]	1 (16.7) [7]	0	2 (33.3) [8, 9]	0	3 (50) [7, 10, 13]	3 (50) [10, 11, 13]
Discrete event simulation	7 (87.5)	1 (14.3) [12]	1 (14.3) [13]	6 (85.7) [7–11, 13]	4 (57.1) [9, 10, 12, 13]	0	3 (42.9) [7, 8, 11]	2 (28.6) [7, 11]	1 (14.3) [13]	5 (71.4) [8–10, 12, 13]	4 (57.1) [8, 9, 11, 12]	0	4 (57.1) [7, 10, 12, 13]	3 (42.9) [8, 9, 12]	0	2 (28.6) [11, 13]	4 (57.1) [7, 10, 11, 13]
Agent-based models	2 (25)	0	0	2 (100) [8, 13]	1 (50) [13]	0	1 (50) [8]	0	0	2 (100) [8, 13]	1 (50) [8]	0	1 (50) [13]	1 (50) [8]	0	1 (50) [13]	1 (50) [13]
System dynamics	5 (62.5)	0	5 (100) [8–11, 13]	0	2 (40) [9, 13]	3 (60) [8, 10, 11]	2 (40) [10, 11]	1 (20) [11]	0	4 (80) [8–10, 13]	5 (100) [8–11, 13]	0	0	2 (40) [8, 9]	0	3 (60) [10, 11, 13]	3 (60) [10, 11, 13]
Compartmental models	2 (25)	1 (50) [6]	1 (50) [8]	0	1 (50) [6]	1 (50) [8]	1 (50) [8]	0	0	2 (100) [6, 8]	2 (100) [6, 8]	0	0	2 (100) [6, 8]	0	0	0

^a^Denominator out of 8 (total number of decision frameworks identified).

^b^Denominator out of the number of frameworks that have discussed that specific modelling approach (i.e. second column).

For traditional modelling approaches, an even greater degree of disagreements was observed in how structural features were specified. Most frameworks did not discuss the notion of resource constraints for decision trees. Of the frameworks that describe the dimension of time and interactivity, they were consistent in characterizing decision trees as static, fixed time horizon (i.e. untimed) models. However, for the remaining two structural features (i.e. population resolution, first-order uncertainty), less clarity emerged. For Markov cohort models, as per its name, the frameworks all agreed that this modelling approach is not an individual-level modelling approach but rather focused at the aggregate-level. Markov cohort models were considered not capable of handling interaction or resource constraints in most except in two of the frameworks (Table 5) [7, 12].

## Discussion

Despite the prevalence in the use of traditional modelling approaches to conduct health economic evaluations, these frameworks all highlight the need for alternative modelling approaches under certain circumstances. For instance, discrete event simulation permits explicit incorporation of queuing theory and may be suitable if the question partly involves resource constraints. Agent-based models, on the other hand, can integrate agent-to-agent interactions and are thus suitable when behavior is considered an important characteristic with the problem at hand (e.g. infectious disease modelling). Indeed, it may be safe to extend that there is no single modelling approach that is capable of answering all types of research questions. HTA agencies and other policy organizations that rely on economic modelling to guide reimbursement and resources allocation decision-making must therefore develop the capacity to construct and critically appraise models outside of what is considered the traditional modelling approaches.

Although several frameworks have been published to distinguish between modelling approaches, there is no clear over-arching or universally-accepted one. Each framework has, in fact, highlighted different selection criteria that may be of importance when choosing the most-suitable approach. A recurring theme that emerged across these frameworks is the necessity for the approach to reflect the underlying theory of the health condition and the characteristics of the health technologies being compared. The modelling approach selected should align with the purpose of the model and the level of detail desired with minimal complexity [4].

However, Table 5 highlighted a concerning observation: there is a general lack of agreement between the decision frameworks on how the structural features specific to each modelling approach are described. This suggests that, by using different frameworks, one may come to a different decision on what constitutes the most appropriate modelling approach. For instance, consider a model that is interested in exploring the cost-effectiveness of therapies for lowering blood pressure in patients with essential hypertension in terms of the prevention of cardiovascular and cerebrovascular events. The model aims to simulate a cohort of patients with heterogeneous characteristics and, given the existing understanding of hypertension, the model must capture the impact of different risk factors on the development of clinical events as these risk factors evolve over time. Additional factors to consider for this decision problem is that resources will be assumed unlimited and that time will be handled discretely. Employing the six generic frameworks without consideration of the practical constraints, we find that four frameworks [7, 9–11] advise for a Markov microsimulation, one framework [13] recommends a Markov or discrete event simulation without specification on whether the Markov model is an aggregate-level or an individual-level model (i.e. microsimulation) while the last specifies for a discrete event simulation or a Markov cohort model [12]. Other cases exist of applying these frameworks to a decision problem and encountering different recommendations in terms of which modelling approach would be recommended.

It may be that, in certain cases, these frameworks do not entirely represent what the authors would consider as best practice, but rather what is recommended and accepted practice in the jurisdiction in which they work (i.e. many countries now have national reimbursement bodies that provide guidelines on economic modelling and may influence the choices of how the researchers in those countries developed their framework). Indeed, different frameworks were found to address different sets of modelling approaches (Table 2). It would be expected that frameworks would characterize the structural features specific to a particular modelling approach similarly although this was not observed (Table 5).

Despite this, another consistent recommendation emerged from these studies in that the decision of which modelling approach to select is dependent not only on the structural assumptions but often also on the practical considerations. It is rarely possible to consider one without the other. Even amongst the frameworks that solely incorporated structural features [6, 8, 10, 11], half included a separate discussion on the practical considerations to modelling [8, 10]. The selection of the appropriate modelling approach is therefore iterative. The clinical research question (i.e. characteristics of the disease and its intervention) dictates which structural features are important. This filters down the range of suitable modelling approaches and subsequently, practical elements such as simplicity, computational efficiency, end-user requirements and transparency may impact the decision on the best-suited modelling approach.

One unresolved question remains: the trade-off between simplicity and internal validity. In most of the frameworks and in other broad economic evaluation guidelines, the majority support the notion that the model structure should be kept as simple as possible [13, 16]. Barton and colleagues mention that more complex models are only justifiable when the increased complexity leads to more valid results [9]. Another interpretation to the above recommendation is that, when selecting a simpler technique, a modeller should ensure that any error incurred from omitting certain aspects of the disease and its intervention will not materially bias a study’s results [17]. But, how much simplification is possible without compromising a model’s validity? Unfortunately, this is not a straightforward issue as it is based on several factors including the nature of the decision problem (i.e. clinical condition and the treatment alternatives being modelled) and several practical considerations (e.g. available data, time and budget) [18]. Greater research and education is thus necessary for both modellers and decision-makers to better characterize and understand the implications of such a trade-off.

The observed discrepancy observed between frameworks in the recommendations they provide on which modelling approach is appropriate leads to the question of whether selecting different modelling approaches do in fact impact the model’s results and conclusions? When does it truly matter which modelling approach is used? For instance, to what extent does patient heterogeneity have an impact such that a Markov microsimulation or a Markov cohort model would produce diverging results? Similarly, to what extent does queuing and constrained resources impact the cost-effectiveness of an intervention such that it warrants the need for a discrete event simulation? These frameworks were all found to lack a sufficient evidence-base as most were based on general heuristics. A means to answer the above questions empirically would be to assess a model’s validity. One approach, based on the concepts of cross-validation, would be to compare the results between highly-dependent models that employ different modelling approaches to otherwise address the same research problem by using the same data parameters and sharing common assumptions. Such exercises may inform when it empirically matters whether a particular modelling approach is selected and some of the early pioneers in such activities include the Mount Hood Challenge for diabetes modellers [19].

A recent systematic review was published focused on cross-validation work in health economic models, evaluating the impact of structural features on the choice of the modelling approach [20]. Population resolution was found to have minimal impact empirically as both aggregate- and individual-level models generated nearly identical results. Rather, consideration on this structural feature was relevant in terms of a practical trade-off between validity and feasibility (e.g. individual-level models required fewer simplifying assumptions, thus increasing its face validity but at the expense of being more time- and data-intensive; and vice versa). In terms of the criterion of interactivity, infectious-disease models have consistently showed that, depending on the assumptions regarding the probability of disease exposure, dynamic and static models will produce dissimilar results and lead to opposing policy recommendations [20]. Further research in this area is still required as it may provide the evidence that is necessary to better guide the development of evidence-based decision frameworks.

One challenge that arose over the course of this study was the heterogeneity in the terminologies employed to describe the modelling approaches. For instance, for Markov cohort model, Chick’s [11] framework used the term “finite difference model” while the original framework by Brennan referred to it as “simulated Markov model” [13]. This was even more evident for Markov microsimulation as it was referred to by a wide range of terms including: “individual sampling model” [9, 10], “patients evolve on discrete time grid” [11], “patient-level simulation” [11], “Monte Carlo Markov models” [13], “Monte Carlo simulation/microsimulation” [8] and “First-order Markov model” [7]. This is concerning as continued use of unclear and inconsistent terminologies can hamper communication between modellers and mislead understanding on these frameworks. It is possible (and we acknowledge) that the differences observed between frameworks may not only lie with their recommendations but may also be partly due to differences in their semantics. Given the cross-disciplinary nature of this field, greater effort is necessary to standardize the terminology that is being used. Some excellent work has emerged from ISPOR-SMDM good research practice guidelines [3–5] although much remains to be done.

A limitation with this study is that it focused mainly on literature from the health care context. As previously mentioned, the modelling approaches used in health economic evaluations originated from the fields of mathematics, operations research and industrial engineering. Consequently, a vast and rich source of literature is likely to exist within those fields that have not been included in this study. By not including studies from other disciplines, this paper may not capture the decision frameworks outside of health care. We acknowledge that this is a limitation to this study although it was necessary to restrict the literature search within the field of health to capture the decision criteria that are specifically relevant to the health context.

## Conclusions

To reiterate, the aim of this systematic review was not to propose a new framework that unifies the existing frameworks or to provide support towards a single one. Rather, this review was intended to identify and critically appraise the collection of decision frameworks that are currently available to health economic modellers and their users. Although most were developed independently, at a minimum, all frameworks were found to involve a comparison of the structural features as a means to distinguish between the approaches. Nearly all frameworks considered the criteria of population resolution and interactivity; which may perhaps be indicative as the absolute minimum needed to be considered when selecting a modelling approach. Furthermore, most authors explicitly considered or discussed the practicalities to modelling as part of their framework. Emerging from this review, we find that the process of selecting an appropriate approach for health economic models involves the consideration of multiple criteria. One must not only align the nature of a given decision problem with the structural features of a modelling approach; practical constraints that are context-dependent must further be examined.

Although decision frameworks are intended to provide a systematic and transparent approach in which to pursue the question of which modelling approach should be chosen, this review found a concerning lack of agreement between frameworks in terms of how structural elements are classified. Thus, by employing different frameworks, different recommendations may emerge. In this case, the use of decision frameworks may provide a false sense of confidence that the appropriate methods were employed for the conduct of an economic evaluation and blindly shut down any further debate on the process and the considerations for which a modelling approach was chosen. It is intended that this work will hopefully open dialogue between researchers and policy-makers in terms of providing or requiring greater transparency on how a particular modelling approach is selected. Until better agreement exists amongst frameworks or more empirical research is conducted, we strongly recommend that modellers properly and transparently justify why a particular modelling approach was selected over the others. The choice of a modelling approach is an important and necessary step to any health economic modelling exercise with broad implications on the subsequent model development and evaluation. Given its potential impact on a model’s validity, the choice should be carefully considered, debated and reported.

## Additional files

Additional file 1: Search strategy. Additional file 2: PRISMA 2009 checklist Additional file 3: Level I screening form. Additional file 4: Copies of existing decision frameworks.
