# Supplementary material for: Systematic narrative review of decision frameworks to select the appropriate modelling approaches for health economic evaluations
Source: BMC Res Notes. 2015 Jun 17;8:244. doi: 10.1186/s13104-015-1202-0 (PMC4470071; doi:10.1186/s13104-015-1202-0)
Supplement: Additional file 1: — Search strategy. [file 13104_2015_1202_MOESM1_ESM.docx]

**Additional File 1:** Search Strategy

| Database: Ovid MEDLINE(R) <In-Process & Other Non-Indexed Citations and Ovid MEDLINE(R) 1946 to Present> | |
| --- | --- |
|  | [(Economic evaluation*[Title/Abstract] OR economic outcome*[Title/Abstract] OR economic analys?s[Title/Abstract] OR health economic*[Title/Abstract] OR pharmacoeconomic*[Title/Abstract] or pharmaco-economic*[Title/Abstract])  (37,258) |
|  | (decision* analy* [Title/Abstract] OR model?ng adj (approach* OR method* OR practice* OR technique*)[Title/Abstract]  (33,906) |
|  | (Guid*[Title/Abstract] OR select*[Title/Abstract] OR choos*[Title/Abstract] OR choic*[Title/Abstract] OR categoris*[Title/Abstract] OR framework*[Title/Abstract] OR taxonom*[Title/Abstract] OR recommend*[Title/Abstract]  (4,608,561) |
|  | 1 AND 2 AND 3  (802) |
