# Supplementary material for: Systematic narrative review of decision frameworks to select the appropriate modelling approaches for health economic evaluations
Source: BMC Res Notes. 2015 Jun 17;8:244. doi: 10.1186/s13104-015-1202-0 (PMC4470071; doi:10.1186/s13104-015-1202-0)
Supplement: Additional file 3: — Level I screening form. [file 13104_2015_1202_MOESM3_ESM.docx]

**Additional File 3:** Level I Search Strategy

| 1. Is this study focused on economic evaluation methods?   No (exclude) Yes (include) Maybe (include)   1. Does this study discuss either: 2. frameworks that assist in selecting the approach to modelling?   No (exclude) Yes (include) Maybe (include)   1. criteria or situations in which one should use one modelling approach over another?   No (exclude) Yes (include) Maybe (include)   1. Is this study published in English?   No (exclude) Yes (include) Maybe (include)  NOTE: Markov model, state-transition model, decision tree, discrete event simulation, agent-based modelling, microsimulation, system dynamics modelling |
| --- |
