# Supplementary material for: Systematic narrative review of decision frameworks to select the appropriate modelling approaches for health economic evaluations
Source: BMC Res Notes. 2015 Jun 17;8:244. doi: 10.1186/s13104-015-1202-0 (PMC4470071; doi:10.1186/s13104-015-1202-0)

**Additional File 4:** Copies of Existing Decision Frameworks (Only frameworks that have received publisher’s or author’s permission are presented here). A copy of the framework by Barton et al.(9) and Cooper’s et al. (12) is not presented here.

**Figure 2:** Brennan et al’s classification framework(10). This taxonomy emphasizes the distinguishing structural assumption within each modelling approach and highlights the commonalities and differences between modelling approaches used in health economic evaluation. *(copyright ©2006 Brennan et al, ‘Taxonomy of model structures’ with kind permission from Springer Science and Business Media)*

**Figure 3:** Chick’s framework(11), adapted from Brennan’s taxonomy (copyright ©2007 Chick)

**Figure 4:** Heeg’s radar diagram(7), adapted from Brennan’s taxonomy. The further away from the origin the model’s line crosses the axis of the selection criteria, the better the model is able to incorporate or handle that corresponding structural feature or practical characteristic. (copyright ©2008 Heeg et al, “A radar graph of the strengths and weaknesses of cohort decision trees, cohort Markov models, first-order Markov models and discrete event simulation (DES) models for application in a chronic complex disease such as schizophrenia”, With kind permission from Springer Science and Business Media)

DES: discrete event simulation

**Figure 5:** Stahl’s flowchart to assist in selecting the modelling approach(13). (copyright ©2008 Stahl, “A decision algorithm for choosing a simulation method”. With kind permission from Springer Science and Business Media)

DEEPP: describe, evaluate, explore, predict and persuade; KISS: keep it simple stupid; DES: discrete event simulation

**Figure 6:** Kim et al’s(8) framework specific to infectious-disease modelling, based on an adaptation of Brennan’s framework(10). (copyright ©2008 Kim et al, “*Classification of (mathematical) model types used in health economic evaluation*.” *With kind permission from Springer Science and Business Media)*

**Figure 7:** Jit et al’s(6) framework primarily distinguished between the instances in which a dynamic or static model would be appropriate in infectious disease modelling. The key decision criterion relates to interaction and whether the force of infection is constant or variable. (copyright ©2011 Jit et al, “Flow diagram showing how the choice of a static model, static model with approximation for herd immunity or dynamic model could be made based on answers to seven key questions” With kind permission from Springer Science and Business Media)

**Figure 2:**


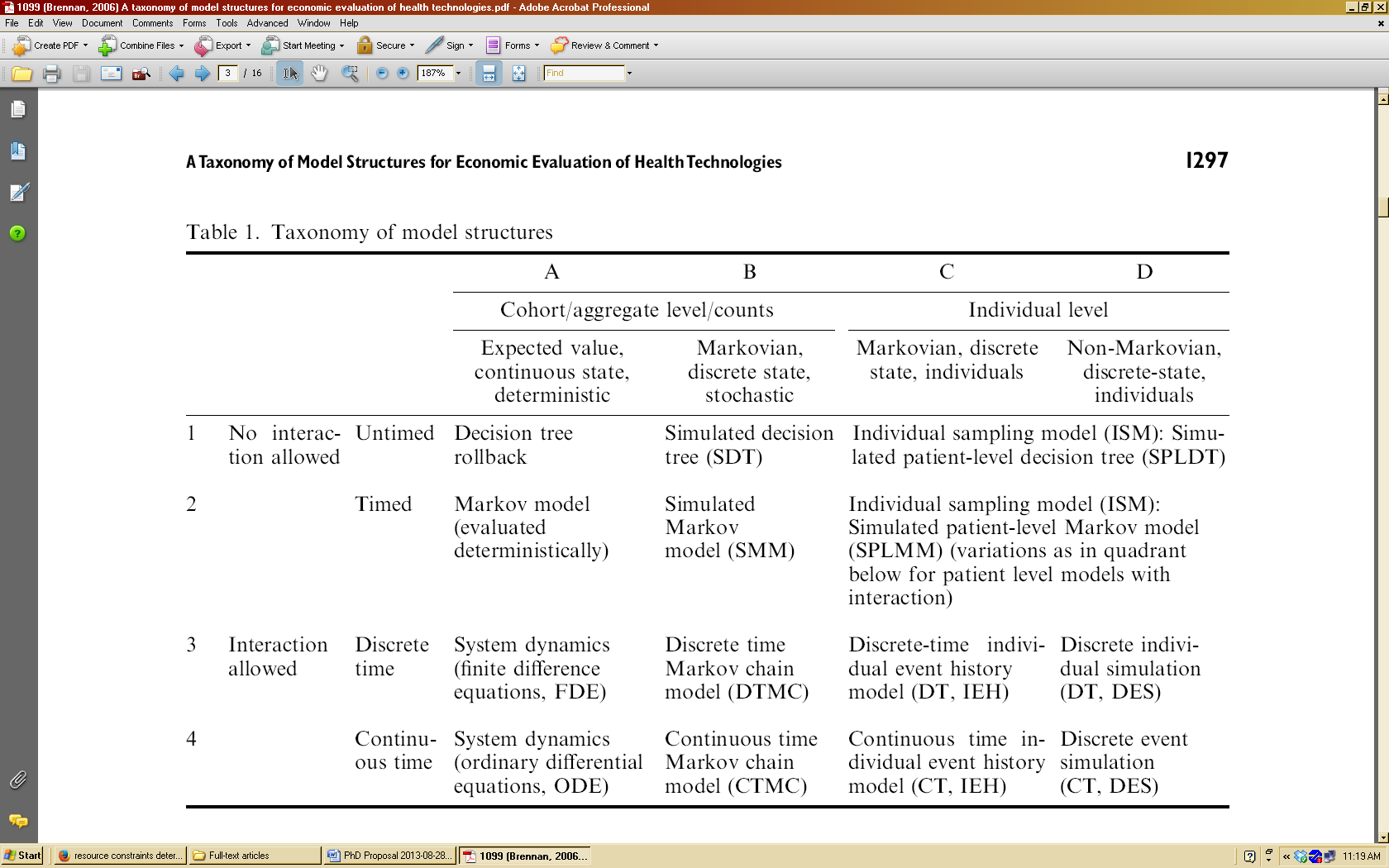


**Figure 3:**


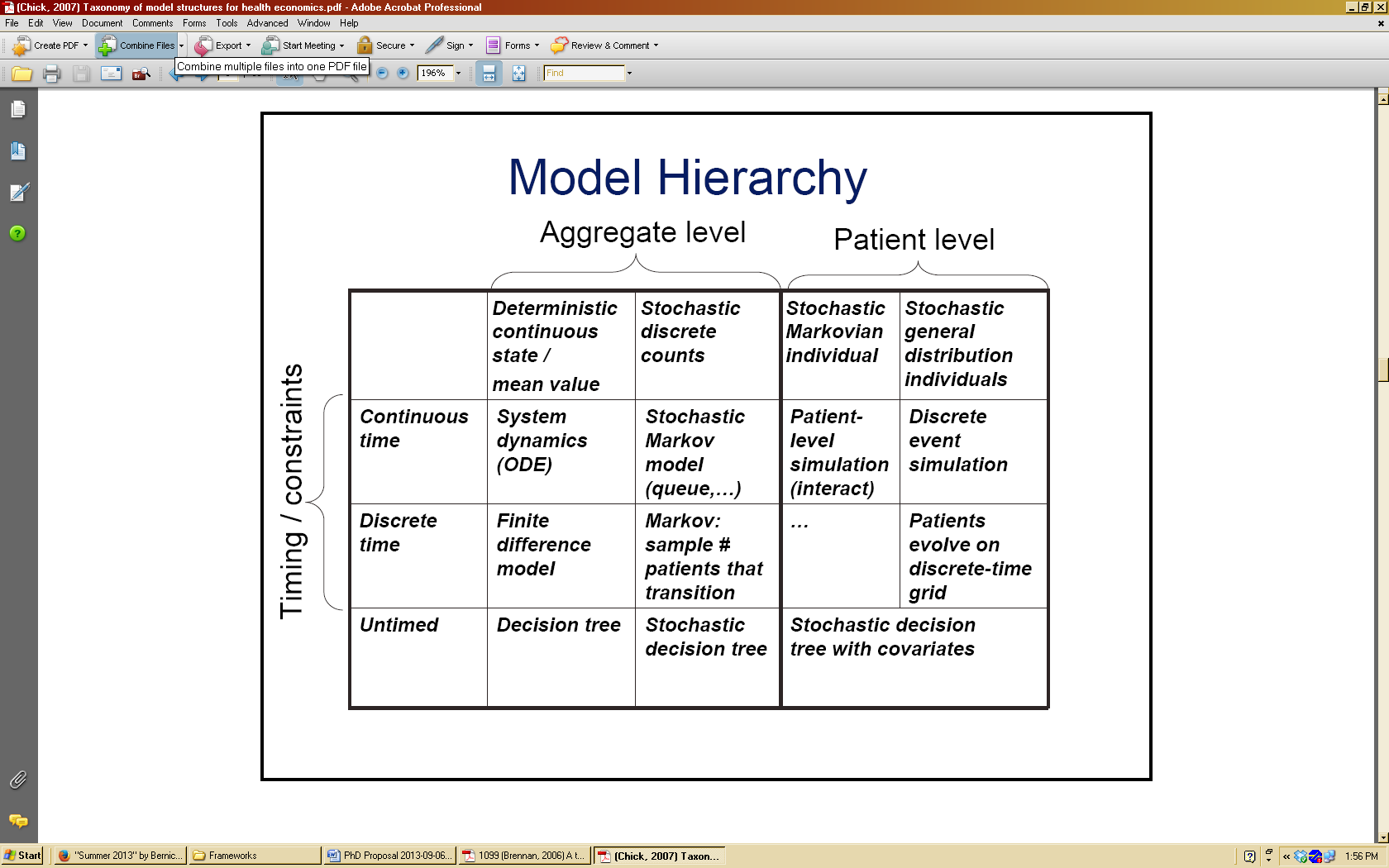


**Figure 4:**


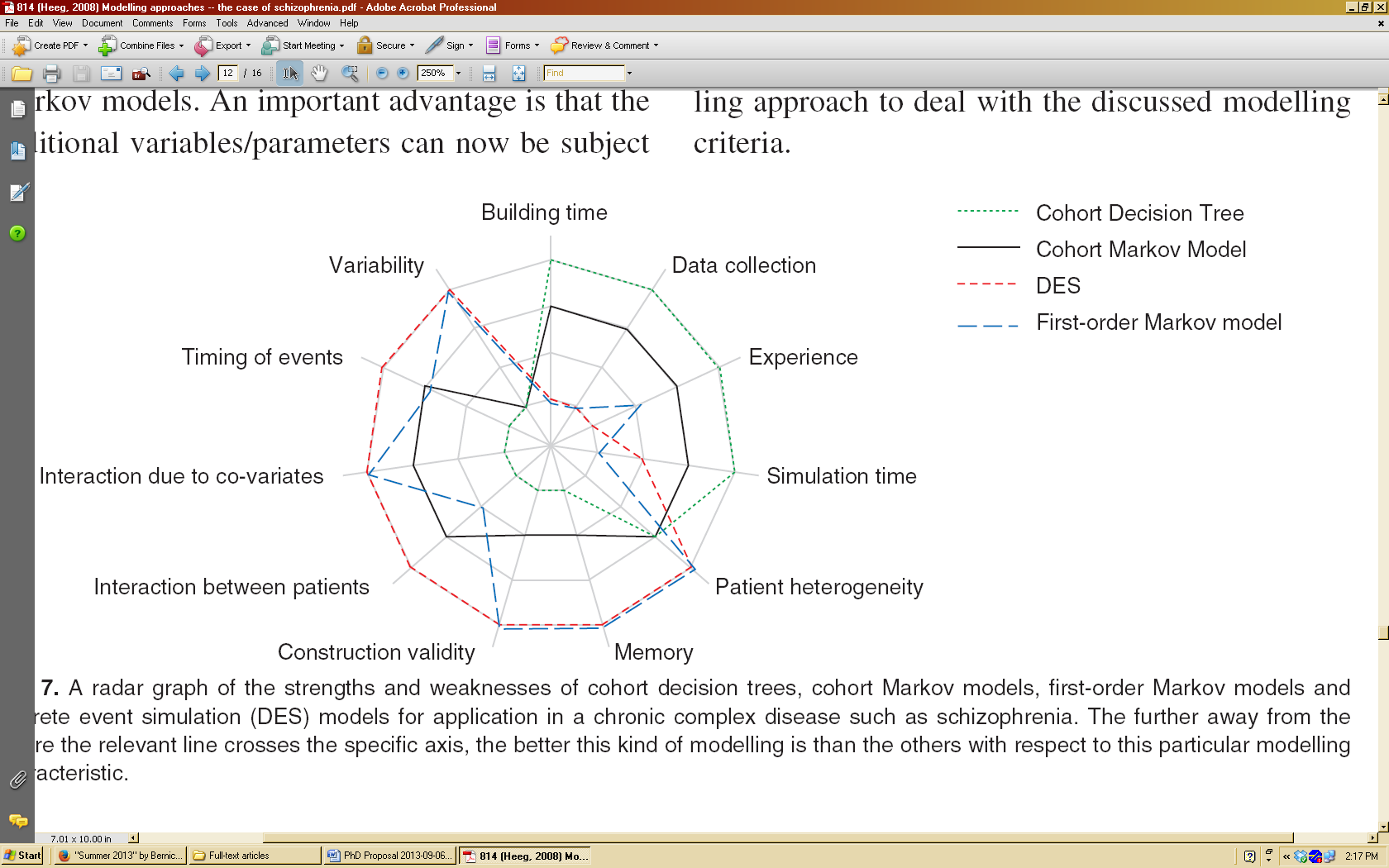


**Figure 5:**

**
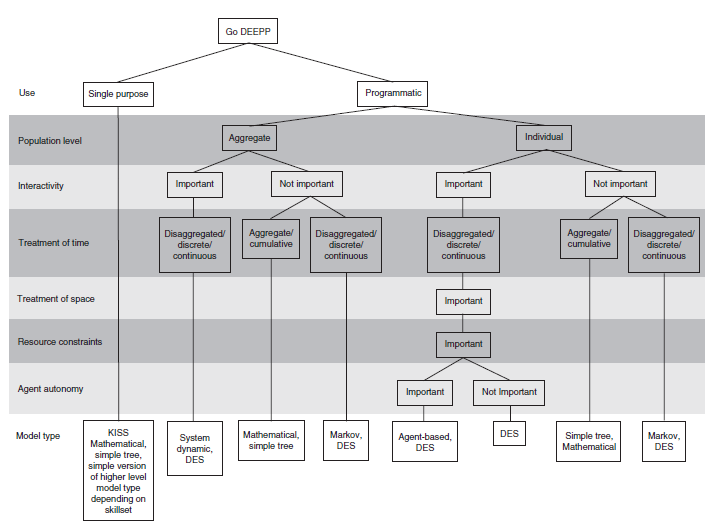
**

**Figure 6:**


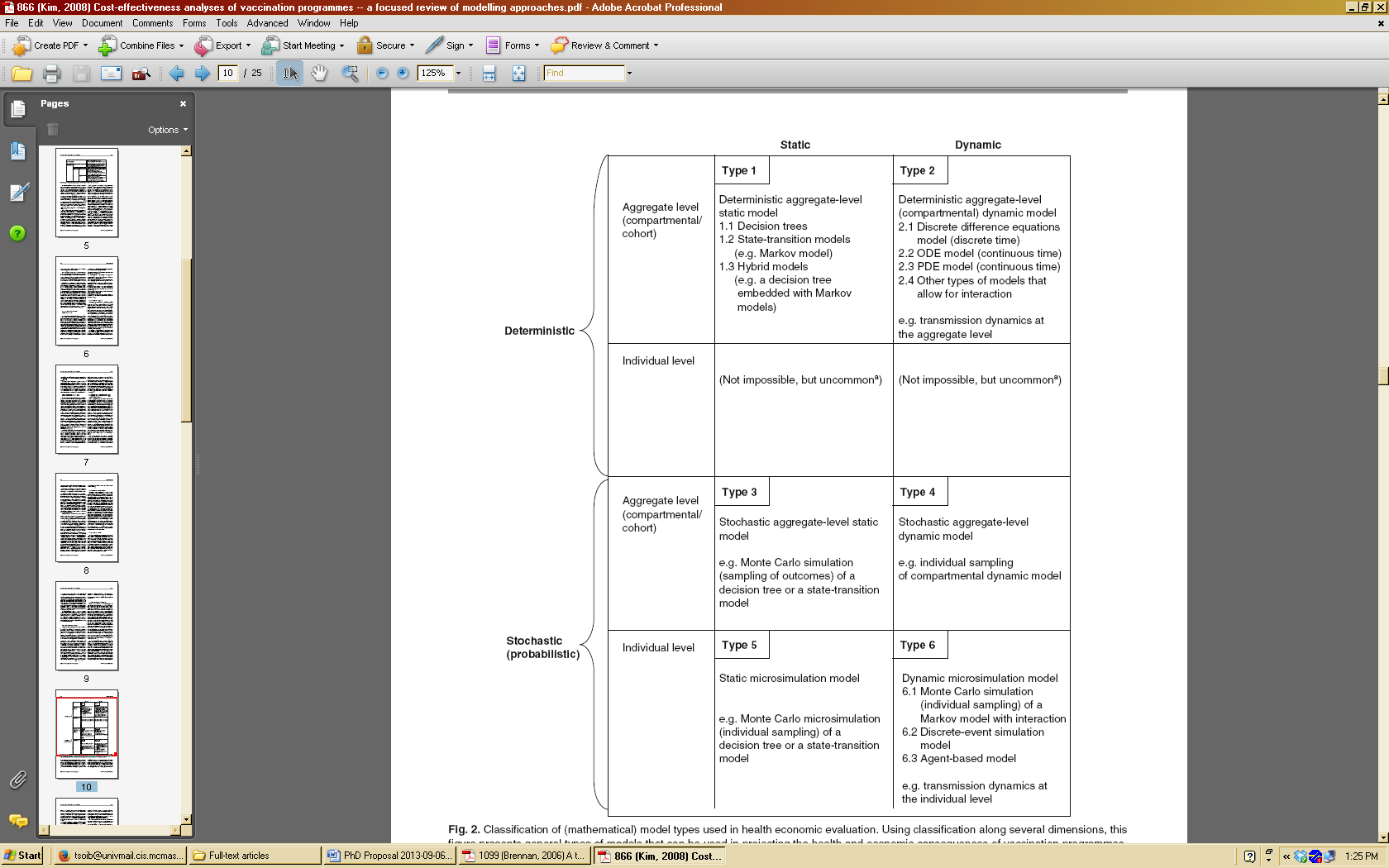


**Figure 7:**


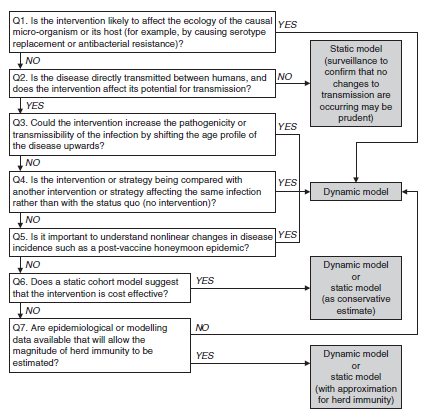

Supplement: Additional file 4: — Copies of existing decision frameworks. [file 13104_2015_1202_MOESM4_ESM.docx]
